# Review of 100 Radicals

**Published:** 1911-12

**Authors:** George F. Cott

**Affiliations:** Clinical Professor of Otology, University of Buffalo; 1195 Main St.


### BUFFALO MEDICAL JOURNAL

VOL. LXVII. DECEMBER, 1911. No. 5

### ORIGINAL COMMUNICATIONS

### Review of 100 Radicals

By GEORGE F. COTT, M.D.
Clinical Professor of Otology, University of Buffalo

AFTER having seen the daily out-put of radical middle ear operations at Vienna in the '90's I made diligent inquiries and found that in America the operation was not performed up to that time. During the next few years however, cases were reported from time to time. I began operating in 1899 and early in 1900 I reported four cases of tympano-mastoid exenteration. Since then I have added a fair quota every year until I now have reached considerably over a hundred. But it is of the first one hundred cases I wish to write in this paper. One has a varied experience in one hundred operations which is well worth the relating. In the first place one cannot always tell what will be found during the progress of the operation.

Occasionally the otologist sees the patient the first or second time, with a vague history only. Temperature having been taken but once a day; patient perhaps in bed; no symptoms relating to disease of the inner ear or a previous attack; many symptoms under-valued by the family; and so you are left to your own resources. Even when one has had a chance to observe the patient for some months the operation findings may surprise you. In a large proportion of cases an almost exact diagnosis can be made. When we have a typic lesion this is generally readily done, but as in many other pathologic conditions there are masked symptoms which often leave one quite in the dark.

When operation is required on a case suffering from some zymotic disease the destructive process is out of all proportion to the symptoms ordinarily found. On the other hand, in a slow long drawn out process the brain may become involved so insidiously that the main lesion will be entirely overlooked.

Occasionally after radical treatment, months or years later, symptoms turn up which make the surgeon think a good long Think. He will occasionally find cholesteatoma develop in the old wound, neuritis, facial paralysis, infection in the internal ear, brain etc. Usually however, after the operation the danger of further trouble is obviated. With all the knowledge of modern treatment of disease of the ear at hand, still there are numberless cases found throughout the country with chronic otorrhoea.

If all such patients were treated early there would be no need of a chronic discharge, always endangering the patient's life.

Let me call to the mind of practitioners and patients the dangers of running ears. First, there is always a drain and sometimes retarded development of the patients: Society shuns them on account of the odor emanating from the diseased bone. There is always danger of infecting some other part of the anatomy. Pus may form under the dura mater, brain abscess, sinus thrombosis, pachymeningitis and lepto-meningitis and labyrinthin infection. These secondary conditions are not at all uncommon. My own experience has given me forty-two cases out of one hundred. In all of these one hundred cases there were but three acute suppurations calling for radical treatment and of course needed hurried attention. These latter are sometimes more destructive than those of long standing. When occurring during or at the end of a zymotic disease the resisting power of the body is away below par, then great damage is done without proportionate symptoms. This is usually not the case in chronic otorrhoea. The amount of damage in an ordinary average case of chronic otorrhoea can never be estimated until the operation is completed. Epidural abscess is often found when least expected. Pachymeningitis is most common and even abscess of the brain has been found occasionally. In one patient one entire hemisphere was bathed in pus yet the patient walked without assistance to the operating table three days before death; having had very little temperature and slight pain in the ear.

The operation is performed first to stop the discharge; relieve the patient from the embarrassing odor which commonly accompanies discharge from the ear; to prevent brain infection and to prevent further constitutional impairment. I believe as people gain more knowledge on this subject there will be but few chronic cases as compared with the many at present, for early treatment is usually successful and then the grave will be robbed of a most fruitful source of revenue.

The percentage of recoveries after a radical operation is large, but few ears continuing to discharge after some weeks. But even when some discharge remains, the danger of intracranial involvement is generally obviated. The one danger however when discharge continues, is cholesteatoma and once in a while labyrinthitis.

The operation itself is not so very simple. One must avoid injuring the lateral sinus, the dura, the internal ear, the facial nerve, etc. Yet all diseased bone must be removed even though these structures are made to suffer, or further infection will result.

The variety of conditions which I found either before or during operation in these one hundred cases of radical middle ear exenteration are interesting and cover a large field. Pus under the dura stands high, but if uncomplicated, all recover.

Epidural abscess, 18; 17 recovered; circumscribed pachymeningitis, 3 recovered; lepto-meningitis, 2 died; sinus thrombosis, 5 recovered; cerebral abscess, 3 died; ulceration of brain, 1 died; band of fibrus adhesion, 1 recovered; fatty thrombus, 2 died; sinus in brain, 2 recovered; cerebellar abscess, 1 died; pus in lateral sinus, 1 recovered; unknown, 3 died; labyrinthitis, 6, 4 recovered; tuberculosis of the ear, 3 died; carcinoma of the ear, 1 died.

The above series of cases of complications mostly within the skull shows a recovery of 33 out of 52 patients or 63.4 per cent. Cases were not selected, in fact some reported here were in extremis when operated upon. This table shows conclusively the dangers of neglecting a "running ear." A large percentage showing some form of trouble within the cranial cavity.

This condition of things can only be remedied when the family physician insists upon proper care being given young patients with acute middle ear suppuration especially following zymotic diseases, for those are most apt to become chronic.

These fifty-two cases of complications were not in as many separate patients, for several with brain abscess and sinus thrombosis had also epidural abscess, each finding is separately classified. Tuberculosis of the ear had not involved the brain at time of operation but patients died some months later of general infection. Sarcoma was found at the operation but cannot be classed as intracranial. Those cases classed as unknown were out-of town operations without any history except chronic otorrhea of very long standing exhibiting no symptoms but lassitude and headache.

Of the labyrinthin cases two recovered with operation, two without operation and two were operated upon with the idea of relieving the patients who really had already developed lepto-meningitis. Pachymeningitis was found three times without other complications after removing the tegmen. Sinus thromboses were typic cases, one exhibiting a temperature varying nine degrees in twenty-four hours; reaching 107½ degrees; the next highest was 106½. In the first the Jugular was tied. During the following year the patient aged 19, had abscesses form in different parts of the body but finally recovered. In one case a fibrous band tied the dura down to the skull and was one-fourth inch in length, nothing could be found to cause the pain of which the patient complained. She got considerable relief after it was excised. Fatty thrombosis gave no symptoms during life and was only found at operation, both patients were in bad condition at the time. A sinus was found in two cases in which a probe entered into the white substance, an inch. One patient died a year later of epidemic cerebro-spinal meningitis through a small opening in the dura ⅛ inch in diameter. Post-mortem showed no connection with old lesion which had completely healed. The second case was operated upon four times within a period of ten years the last time an opening one-half inch was found in the brain six years after the third operation, in which the tip of a finger could be placed. She made a good recovery. Cerebellar abscess was found post-mortem. Pus in lateral sinus was found in a case of thrombosis walled off at either end of the lateral sinus, patient made a good recovery.

Facial paralysis has been rather too frequent. I certainly have had 15 per cent. follow the operation. One came on during the operation. All the rest followed several hours to several days later. All recovered in four weeks to several months without any treatment.

### METHOD OF OPERATING.

When sclerosis affects the whole mastoid I open the mastoid portion only enough to give free drainage to antrum and middle ear, opening all small spaces in the antral wall, removing outer wall of aditus and external wall of epitympanicum. If on opening the tegmen is found, this is thoroughly explored and freely opened. Whenever diploic mastoid is encountered all cells are laid bare and the antrum opening made correspondingly larger. The whole cavity is then covered with periosteum and skin and the ear drained through the natural opening. Healing takes place from four weeks up. Some discharge indefinitely, others intermittently, for some time. A few begin after some years but are easily controlled. All however receive great benefit. Since I mildly curette the bony eustachian tubes, recurrence is less often. Healing is promoted by using the Siebenman flap which prevents granulations from forming at opening of the meatus. It consists of slitting up the soft meatal wall and at its outer portion cutting a "V," the base upwards, and stitching the flaps backwards. Immediate skin grafting has not been successful in many hands. My patients receive no skin preparation before operation. No hair cut or shaving the field. Iodine only is used in 5 per cent. tincture. Healing is by first intention with few exceptions. This method I have used the last two years and shall continue it hereafter.

1195 Main St.

